# Fostering diagnostic competence in prospective mathematics elementary teachers through epistemic activities

**DOI:** 10.3389/fpsyg.2023.1285919

**Published:** 2023-12-21

**Authors:** Jan Philipp Volkmer, Andreas Eichler, Elisabeth Rathgeb-Schnierer

**Affiliations:** Department of Mathematics and Natural Science, University of Kassel, Kassel, Germany

**Keywords:** diagnostic competence, diagnostic thinking, fostering diagnostic competence, epistemic-activities, prospective elementary teachers

## Abstract

Research on fostering teachers’ diagnostic competence and thinking has become increasingly important. To this end, research has already identified several aspects of effective fostering of teachers’ diagnostic competence. One of the aspects is assignment of the role as a teacher in interventions but, so far, assignment of the role of student has hardly been considered. Based on a model of the diagnostic thinking process, this paper operationalizes the role of the student by solving specific tasks and the role of the teacher by analyzing student solutions. Furthermore, based on previous research, it is assumed that assigning both roles is effective in promoting diagnostic competence. The following research addresses the development of 137 prospective teachers’ diagnostic thinking in an experimental pre-post-test study with four treatment conditions, which vary prospective teachers’ working with tasks and students’ solutions to those tasks. The quantitative results show that a treatment integrating focus on tasks and students’ solutions is equally as effective as a treatment focusing solely on students’ solutions, and also that a treatment focusing solely on tasks has no effect.

## 1 Introduction

**Figure 1:**
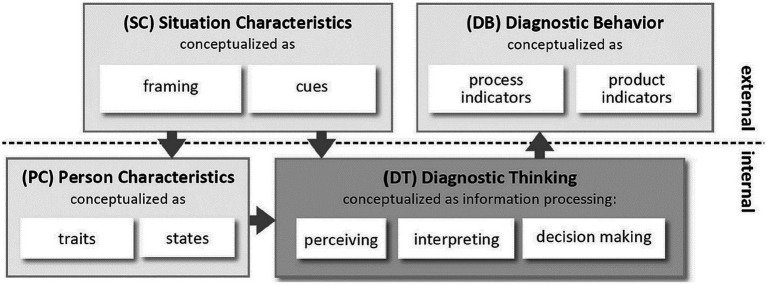
Framework of Loibl et al. (2020, p. 1).

Formative assessment of students’ knowledge, abilities, or learning processes is a crucial task for teachers (Behrmann and Souvignier, 2013; Herppich et al., 2018; Chernikova et al., 2020; Loibl et al., 2020). Teachers’ ability to deal with situations of formative assessment is often called teachers’ diagnostic competence (Binder et al., 2018). A framework that allows the conceptualization of different situations, in which teachers rely on their diagnostic competence and (as a core element of this model) apply diagnostic thinking, is provided by Loibl et al. (2020). In this framework (Figure 1), diagnostic thinking (DT) is influenced by situation characteristics (SC) and person characteristics (PC). The result of diagnostic thinking is called diagnostic behavior (DB).

One frequently-addressed diagnostic situation in educational research concerns the accuracy of teachers’ judgment, involving teachers’ ability to estimate task difficulty in relation to students’ ability (Anders et al., 2010; Südkamp et al., 2012; Schrader, 2013; Ostermann et al., 2018). However, another diagnostic situation has become increasingly important in recent years, namely, the assessment of students’ learning processes. Compared to the accuracy of judgment, assessing students’ learning processes is considered a more authentic task for teachers (Leuders et al., 2018; Enenkiel et al., 2022; Kron et al., 2022).

In this paper, we address teachers’ abilities to assess students’ learning processes and refer to the framework of Loibl et al. (2020; *cf.* also Leuders et al., 2018) who built on the general model of teachers’ competencies proposed by Blömeke et al. (2015), aiming to differentiate “between teacher dispositions relevant for a specific professional competence, diagnostic activities that are carried out in a specific situation, and the performance resulting from these dispositions and activities in a specific situation” (Codreanu et al., 2021, p. 2). Another reference is made to a model of diagnostic thinking based on the work of Nickerson (1999), which differentiates two major thought processes: (1) working with a task to develop a default model of a potential student’s solution and (2) judging specific solutions by students based on epistemic activities (Eichler et al., 2022). Although Nickerson’s model (1999) originally refers to face-to-face communication, the approach is also used to model diagnostic competence (Philipp, 2018). A third reference is made to an approach in medicine, clinical reasoning, which could be understood as constituted by so-called epistemic activities (Kiesewetter et al., 2016; Heitzmann et al., 2019). In this paper, we combine the model of Nickerson (1999) and the set of epistemic activities (Chernikova et al., 2020) to describe teachers’ diagnostic thinking.

Research suggests that it is both necessary (Stahnke et al., 2016) and possible to promote (prospective) teachers’ diagnostic competence (e.g., Klug et al., 2016; Herppich et al., 2018). Particularly, recent research has yielded promising training strategies to foster teachers’ diagnostic competence referring to video vignettes (e.g., Enenkiel et al., 2022), simulation of diagnostic situations (Schons et al., 2022) or exploring students’ solutions (Eichler et al., 2022; Philipp and Gobeli-Egloff, 2022). However, it is still necessary to investigate promising conceptualizations of interventions aiming to promote teachers’ diagnostic competence (e.g., Chernikova et al., 2020).

In this paper, we contribute to the question of whether conceptualization of an intervention is effective in promoting teachers’ diagnostic competence in terms of their diagnostic thinking (DT). Based on Nickerson’s model, we differentiate between (1) working with an open-ended arithmetic task to develop a default model of a potential student’s solution and (2) judging specific solutions from students based on epistemic activities. Our main research question is: which of the two aspects of diagnostic thinking is more effective in an experimental pre-post-test design. The study includes different treatment groups of prospective teachers focusing either on the prospective teachers’ own solutions or on the solutions of elementary school students. Based on previous research, both foci are assumed to be effective for promotion of diagnostic competence. In the following sections, we refer to the theoretical underpinnings of our study and present findings that suggest focusing on student solutions as the most promising strategy for promoting prospective teachers’ diagnostic thinking as part of diagnostic competence.

### 1.1 Diagnostic thinking

In the framework for teachers’ diagnostic competence of Loibl et al. (2020), diagnostic thinking (DT) represents the center, but is not further conceptualized. For conceptualizing diagnostic thinking by assessing students’ written solutions, the model from Nickerson (1999) has already been used with promising results (Ostermann et al., 2018; Philipp and Gobeli-Egloff, 2022).

**Figure 2:**
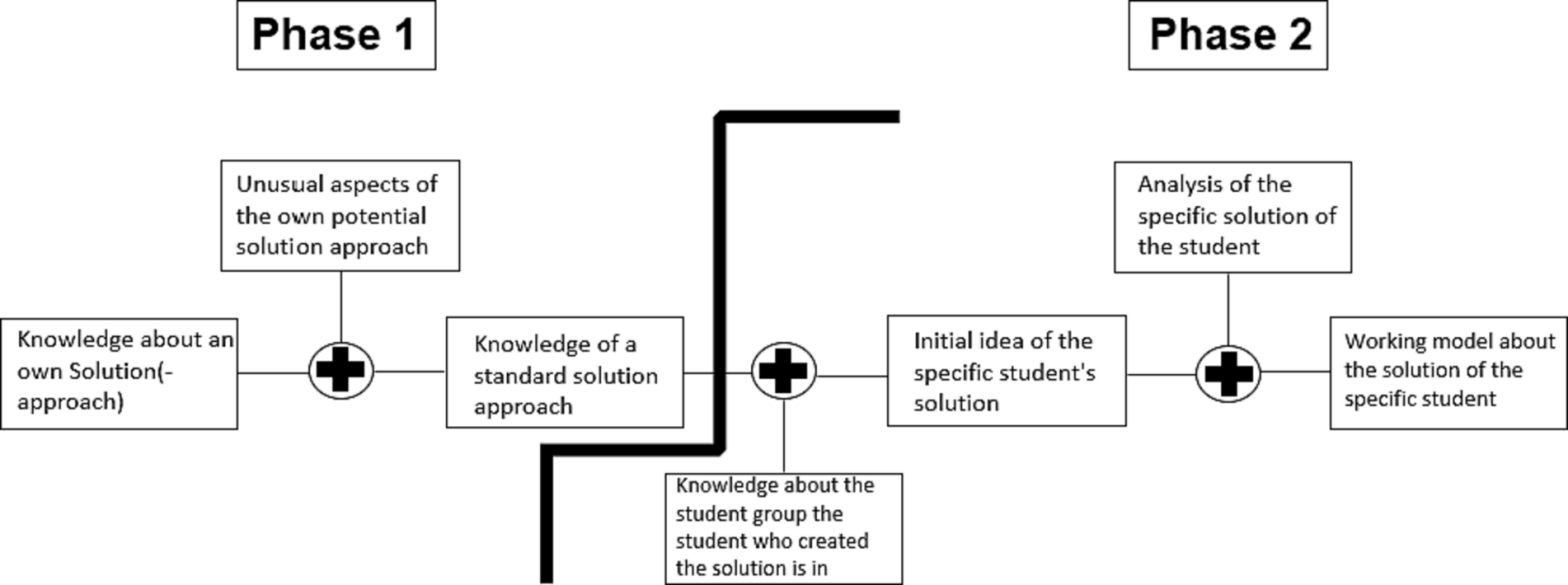
Adaption of the model of specific others’ knowledge (Nickerson, 1999).

Nickerson (1999) designed his model for face-to-face communication and assumed that the process of creating a model of specific others’ knowledge starts with the diagnosing person’s own knowledge. The diagnosing person is aware of his or her specific knowledge and, by narrowing down that knowledge, arrives at a standard model for the knowledge of random others. This default model is further specified by matching it with specific information or experience regarding a group of specific others. Finally, this initial model of specific other people’s knowledge is compared to the information obtained on an ongoing basis, which leads to a working model of a specific person’s knowledge. The model of Nickerson (1999) is adaptable to diagnostic thinking (Philipp, 2018; Loibl et al., 2020) in a diagnostic situation, in which students’ written solutions have to be assessed, as shown in Figure 2: When a teacher makes a judgment about a student’s solution to a particular task, he/she might first think about his or her own solution. He/she has to adapt this solution approach, which is a potentially more complex solution than a student’s solution and, as a consequence, develops a standard model of a random student’s solution (phase 1). Before analyzing a specific student’s solution, the teacher might refer to additional information about the group of students to which said student belongs, such as the student’s grade level. He/she then uses this knowledge to anticipate a possible solution for the specific student, considering that a fourth grader might produce a different solution than a tenth grader. After this step, he or she goes through the student’s solution step-by-step, continuously processing the information, and as a result, gains a working model of the solution of specific students (phase 2).

Concerning phase 2 of Nickerson’s (1999) model, the development of a working model of a specific student’s solution (see Figure 2) can be refined by an approach of clinical reasoning (Fischer et al., 2014; Chernikova et al., 2020). Clinical reasoning was also proposed by Leuders et al. (2018) as one possible conceptualization of diagnostic thinking. Understanding clinical reasoning as a model of diagnostic thinking, Fischer et al. (2014) distinguished epistemic-diagnostic activities (*cf.* also Heitzmann et al., 2019; Table 1). These activities are related to perceiving and interpreting, which are crucial aspects of diagnostic thinking in the model of Loibl et al. (2020) (Table 1, left side). For example, the epistemic activity of “identifying a problem” is a form of “perceiving.” Likewise, the epistemic activities “questioning” and “generating hypotheses” can be understood as interpreting activities. Therefore, the epistemic-diagnostic activities are appropriate to describe perceiving and interpreting as two aspects of diagnostic thinking (Eichler et al., 2022). As Table 1 shows, the generally formulated epistemic activities were adapted for the project and assigned to aspects of diagnostic thinking.

**Table 1:** Epistemic activities as elements of diagnostic thinking.

Aspects of diagnostic thinking (Loibl et al., 2020)	Epistemic activity (Fischer et al., 2014; Chernikova et al., 2020)	Adapted activity
Perceive	Problem identification	Identify manifest (directly observable) characteristics in a solution
Interpret	Questioning	Question manifest characteristics and explore causes
Interpret	Generating hypothesis	Form assumptions about students’ thoughts, reasoning and competencies
Perceive and interpret	Evidence generation/questioning	Back hypotheses, e.g., with manifest characteristics
	Evaluating evidence	Evaluation of the established hypothesis

### 1.2 Promoting diagnostic thinking

The systematic literature review on empirical studies of Stahnke et al. (2016) revealed that prospective teachers struggle with diagnostic thinking in terms of perceiving and interpreting students’ work, but highlighted that promising approaches exist to develop training methods for the diagnostic thinking of (prospective) teachers. Recently, Chernikova et al. (2020) published a meta-study that includes research on promoting diagnostic competence among teachers and physicians. The meta-study demonstrated a positive influence of problem-solving on the growth of diagnostic competence. According to Chernikova et al. (2020, p. 169), problem-solving with regard to diagnostic thinking is present when “learners received cases/problems and made diagnostic decisions themselves.” This has already been implemented on various occasions. Klug et al. (2016), for example, incorporated independent diagnoses of cases in the form of learning diaries and (Busch et al., 2015) used the fictitious students’ solution. Finally, Besser et al. (2015), Eichler et al. (2022), as well as Schons et al. (2022), relied on authentic digital diagnostic cases that participants work on independently in a learning environment.

One way to support students working on problem-solving tasks that cannot be solved without support is scaffolding (Belland et al., 2017). Chernikova et al. (2020, p. 162) revealed four aspects of scaffolding that support the development of diagnostic competence: “(a) providing examples […], (b) providing prompts […], (c) assigning roles […] and (d) including reflection phases […].” Aside from providing examples, the meta-analysis of Chernikova et al. (2020) reveals that all the other afore-mentioned aspects promote diagnostic competence in mathematical contexts effectively.

Providing examples is present in an intervention “when learners observed modeled behavior, example solutions or worked examples at some time during the training” (Chernikova et al., 2020, p. 169). Although there are studies that integrate providing examples into interventions and demonstrate positive developments of diagnostic competence through the respective intervention (e.g., Besser et al., 2015), the effectiveness of this approach does not seem clear. In their meta-study, Chernikova et al. (2020) could not prove a significant influence of the provided example on diagnostic competence. This goes along with Chernikova et al.’s (2020) call to examine the effect of scaffolding on different situations in which diagnostic competence is needed.

Another aspect of scaffolding in the meta-study of Chernikova et al. (2020) is the provision of prompts. These are divided into “during,” “after” and “long-term” categories, with reference to the time at which the respective prompt is provided. “During” means that the prompt is provided during the diagnostic process. “After” means that the prompt is provided after the diagnostic process, and “long-term” means an ongoing interaction of prompts and diagnostics during an intervention. The meta-study revealed positive effects of all three types of prompts. For example, Philipp and Gobeli-Egloff (2022) proved the positive effect of an intervention with an interplay of prompts on different knowledge areas, including content knowledge, task-specific requirements, and the diagnostic potential of tasks. With regard to diagnostic thinking, prompts can refer to developing knowledge about a task solution by a random student, based on a teacher’s own solution to the task (Phase 1, Nickerson, 1999) or can refer to developing a working model of a specific student’s solution based on epistemic activities (phase 2, Nickerson, 1999).

Assigning the role as a diagnosing teacher is included in almost all studies on the development of mathematics teachers’ diagnostic competence (Gold et al., 2013; Besser et al., 2015; Busch et al., 2015; Klug et al., 2016). In only a few studies is assigning the role of a teacher in real lessons also present (e.g., Besser et al., 2015). However, almost no study includes the role of the student. This means, for example, that (prospective) teachers in interventions do not have to explicitly work on the tasks themselves for which they assess student solutions. Nevertheless, Chernikova et al. (2020) hypothesize a positive effect of assigning the role as student in interventions, as it can be assumed that, in doing so, certain competencies or specific knowledge are acquired. This hypothesis is also supported by the fact that taking the student perspective is understood as part of the diagnostic process (e.g., Philipp, 2018). In relation to the diagnostic thinking framework used in this paper (Nickerson, 1999 and Figure 2), it can also be assumed that the assigning of both student role and teacher role is effective in an intervention to promote diagnostic competence. This is based on the two phases shown in Figure 2. Whereas phase 1 of the thinking process focuses strongly on a prospective teachers’ own solution to the underlying task, thus addressing the role as student, phase 2 focuses on the specific student solution to the task, thus addressing the role as teacher. In the present study, the prospective teachers are assigned the role of a task-solver (student) or the role of a diagnostician (teacher) in different interventions.

Reflection phases also have a significant influence on the growth of diagnostic competence (Chernikova et al., 2020). Chernikova et al. (2020) reported that people with higher prior knowledge benefit more from reflection phases than those with lower prior knowledge. Furthermore, a positive effect of collaborative phases was identified for studies in teaching.

Existing studies include aspects of improving teachers’ diagnostic competence that have been shown to be effective: a problem-solving approach and scaffolding. However, there is a lack of studies that design treatments explicitly oriented toward varying specific aspects of scaffolding. In this research, we explicitly refer to aspects of diagnostic thinking that focus on phase 1 and phase 2 according to the adapted and refined model of Nickerson (1999) and vary the aspect of assigning roles systematically.

### 1.3 Conclusion, research questions and hypotheses

The research presented in this paper is based on a model of diagnostic thinking that includes two phases according to Nickerson (1999): (1) working with an open-ended arithmetic task for developing a default model of a potential student’s solution and (2) assessing specific solutions from students based on epistemic activities. These two phases refer to roles that prospective teachers adopt: the role of a student (1) and the role of a teacher (2). We assume that improving prospective teachers’ diagnostic thinking as part of their diagnostic competence needs to address both phases of diagnostic thinking and, respectively, both roles as a student and as a teacher in an intervention. Based on this model and the resulting assumption, our main research question is the following:

RQ1: How do interventions that address the two phases of diagnostic thinking affect prospective teachers’ epistemic activities?

The main approach of this study is to investigate this research question with an experimental pre-post design, in which we alternate focus between the two phases of diagnostic thinking. In this design, we compare four conditions of intervention:

– The first condition (C_1_-task) addresses the first phase of teachers’ diagnostic thinking, where the prospective teachers take the role of students and develop their own approach to open-ended arithmetic tasks in order to develop a default model of (potential students’) written solutions to open-ended arithmetic tasks.
– The second condition (C_2_-student) addresses the second phase of teachers’ diagnostic thinking, where the prospective teachers work on actual students’ written solutions to open-ended arithmetic tasks and thus take the role of teacher.
– The third condition (C_3_-integrated) addresses both phases of diagnostic thinking, and thus the whole process of diagnostic thinking, which includes a default model of solutions and work on actual students’ written solutions.
– The fourth condition (C_0_-control) is a control condition with no reference to diagnostic thinking.

This leads to a 2×2 design by differentiating between working with open-ended arithmetic tasks (yes/no) and judging students’ solutions to these tasks (yes/no).

Based on the model of diagnostic thinking, we assume that condition C_3_-integrated is the most promising approach to improve prospective teachers’ diagnostic thinking since this condition addresses both phases of diagnostic thinking. Concerning the conditions C_1_-task and C_2_-student, we assume that working with specific students’ solutions is a more authentic situation for prospective teachers than working with a task (Südkamp et al., 2012). For this reason, we assume that condition C_2_-student is more promising to foster prospective teachers’ diagnostic thinking than condition C_1_-task. However, we also assume that teachers’ own work with tasks and their potential solutions or difficulties contributes to their own diagnostic thinking. Thus, we assume that condition C_1_-task is more effective in improving teachers’ diagnostic thinking than condition C_0_-control. Therefore, we refer to the following hypothesis concerning RQ1 (How do interventions that address the two phases of diagnostic thinking affect prospective teachers’ epistemic activities?).

*H1*: To improve teachers’ diagnostic thinking, C_3_-integrated is more effective than C_2_-student, C_2_-student is more effective than C_1_-task and C_1_-task is more effective than C_0_-control.

Some results in educational research imply that a specific order of conditions in an intervention is more promising than other orders (Harr et al., 2015). For this reason, we address the question of whether a specific order of conditions addressing the previously-discussed two phases of diagnostic thinking is more promising than other orders in a second experimental phase:

RQ2: Is a specific order addressing the two phases of diagnostic thinking in an intervention more effective in improving prospective teachers’ diagnostic thinking than other orders?

Specifically, we analyze whether one of the orders C_1_-task-C_2_-student, C_2_-student-C_1_-task or C_3_-integrated-C_3_-integrated is more effective in improving prospective teachers’ diagnostic thinking than the other orders. We investigate RQ2 without a directed hypothesis.

The approach to measuring prospective teachers’ diagnostic behavior as an observable expression of the quality of prospective teachers’ diagnostic thinking refers to the amount and variety of prospective teachers’ epistemic activities when diagnosing written solutions provided by students. We describe the measurement and intervention in the following section.

## 2 Materials and method

### 2.1 Treatments

**Figure 3:**
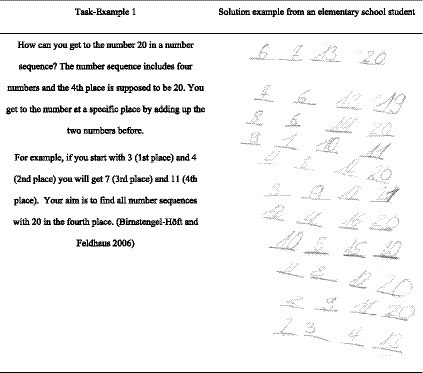
Example of an open-ended mathematical task and a students’ solution.

We developed three different treatments A (for condition C_1_-task), B (for C_2_-student), and AB (for C_3_-integrated). The treatments are based on proven positive effects on the promotion of diagnostic competence (Chernikova et al., 2020). One main element of the treatments is represented by open-ended mathematical tasks (see Figure 3). The open-ended mathematical tasks are defined as “tasks that bear the potential to cognitively activate students, allow different starting points, various approaches, and multiple solutions, and enable students with different ability levels to participate and work on the same tasks” (Rathgeb-Schnierer and Friedrich, in press, p. 2; see also: Yeo, 2017). This means the processing level is open enough that they can be solved substantially by students as well as by the prospective teachers in our conditions. In total, we deployed three open-ended arithmetic tasks in all conditions: C_1_-tasks, C_2_-students to C_3_-integrated in different ways. One example of these tasks is listed in (Figure 3).

Each treatment is developed for 7 weeks with one seminar per week lasting 90 min. All treatments are based on a problem-centered learning approach with a focus on open-ended arithmetic tasks. When solving an open-ended task, prospective teachers deal with a case or a problem as per the model of Chernikova et al. (2020). According to Nickerson (1999), this is understood as part of diagnostic thinking. The evaluation of a student’s solution to an open-ended task also represents a case or problem for which prospective teachers activate diagnostic thinking. Therefore, all treatments feature the proven positive effects of a focus on problem-centering and scaffolding (see Table 2).

**Table 2:** Characteristics of treatments A, B, and AB in terms of characteristics of successful interventions for promoting prospective teachers’ diagnostic thinking.

Characteristics of successful interventions	Treatment A	Treatment B	Treatment AB
Problem-centred approach	Prospective teachers solve open-ended tasks as part of diagnostic thinking	Prospective teachers analyse students’ solutions to open-ended tasks as part of diagnostic thinking	Prospective teachers solve open-ended tasks and analyse students’ solutions for these tasks
Providing examples	Examples of a task solution as synthesis of all task solutions of the prospective teachers	Examples of a diagnosis as synthesis of all prospective teachers’ analyses of students’ solutions	Integration of examples of prospective teachers’ task solutions and analyses of students’ solutions
Providing prompts	Prompt referring to main features of open-ended arithmetic tasks and appropriate solution strategies	Prompt referring to competency domains addressed with open-ended tasks and epistemic activities	Integration of both prompts of treatment A and B
Assigning roles	Role of a student, who solves an open-ended task	Role of a teacher, who analyses students’ solutions	Role of student, who solves an open-ended task and a teacher, who analyses students’ solutions
Reflection phases	Reflection by comparing prospective teachers’ solutions to open-ended tasks in pairs and as an entire class	Reflection by comparing students’ solutions to open-ended tasks in pairs and as an entire class	Reflection by comparing prospective teachers’ and students’ solutions to open-ended tasks in pairs and as an entire class

Although Chernikova et al. (2020) could not clarify the effect of providing examples, we decided to integrate examples into the treatments. Concerning treatment A, examples refer to exemplary solutions to the open-ended tasks that were selected from all the solutions provided by prospective teachers. Concerning treatment B, examples refer to exemplary analysis of students’ solutions that were selected from all analyses of students’ solutions. In treatment AB, we integrated the examples of treatment A and B (*cf.* Table 2).

Prompts are information given to learners during the learning process to support it (Chernikova et al., 2020). In treatment A, the prompts contained detailed information about the main features of open-ended arithmetic tasks. To this end, the specific features of these tasks were discussed with the prospective teachers, such as allowing different starting points, allowing different ways of solving problems, and allowing learners with different levels of proficiency to participate. In addition, the prompts included specific information about problem-solving strategies that the prospective teachers used in solving the open-ended tasks. In treatment B, the prompts contained the information that the prospective teachers need in order to consider different content-oriented aspects while assessing a student’s solution. According to Rathgeb-Schnierer and Schütte (2011) possible aspects concerning students’ solutions to open-ended arithmetic tasks are basic arithmetic knowledge, the solution process, or the communication of a solution. Also, the prompts included a description and examples of the epistemic activities (*cf.* Section 1.1) when diagnosing the students’ written solution to open-ended tasks. With the latter part of the prompt, prospective teachers were also encouraged to identify manifest features in students’ solutions and hypothesize about different aspects of the solution. In addition, prospective teachers learned that it is best to support hypotheses with the identified manifest characteristics. In treatment AB, we integrated both prompts (*cf.* Table 2).

In teacher education, the roles of teacher and learner are the most typical (Chernikova et al., 2020). According to Chernikova et al. (2020), assigning these roles has clear positive effects on diagnostic thinking. The treatment groups differ according to the assigned role. In treatment A, prospective teachers are assigned the role of student, solving open-ended arithmetic tasks. In treatment B, prospective teachers are assigned the role of teacher by being asked to analyze students’ solutions to open-ended arithmetic tasks. In treatment AB, prospective teachers are assigned both roles alternately by solving an open-ended arithmetic task and then analyzing students’ solutions to that task (*cf.* Table 2).

All treatment designs additionally include reflection phases that Chernikova et al. (2020) found to be effective for promoting teachers’ diagnostic thinking. These reflection phases occur in all three treatments with a different focus (*cf.* Table 2) and include encouragement to make comparisons. Thus, we explicitly refer to comparison as an activity of effective teaching approaches (Alfieri et al., 2013) and as an integral part of diagnostic thinking (*cf.* Philipp, 2018; Chernikova et al., 2020).

A detailed description of the treatments A, B, and AB is provided in Appendix 1.

### 2.2 Design of the study and sample

To answer the research questions, we designed one experiment with two phases. To answer RQ1, experimental phase 1 was designed. The design of this experimental phase is presented in Table 3. Experimental phase 1 was designed as a pre-post-test study with a sample of 137 prospective teachers who were randomly assigned to one of the four conditions (C_0_-control, C_1_-task, C_2_-student and C_3_-integrated). The prospective teachers were students of the University of Kassel, in the 3rd–5th semester of their 7-semester university studies. All participants had already completed a practical phase at school and followed courses on didactics and science subjects as well as a general course on diagnosing. To minimize the influence factor of a designated teacher, the seminars were held by three teachers on a weekly rotating basis. The treatments A (C_1_-task), B (C_2_-student) and AB (C_3_-integrated) each last one half-semester, i.e., 7 weeks, including 90 min of seminars each week (*cf.* Section 2.1).

**Table 3:** Design and sample of the experimental study.

Experimental phase 1			
	Conditions		
C_1_-task, *n* = 37	C_2_-student, *n* = 40	C_3_-integrated, *n* = 35	C_0_-control, *n* = 25
Pre-test: diagnostic tasks d_1_ and d_2_ (see Section 2.2; all conditions)			
Treatment A: Solving open-ended arithmetic tasks and looking at different solutions from prospective teachers	Treatment B: Analyzing elementary school students’ solutions to open ended arithmetic tasks	Treatment AB: Solving open-ended arithmetic tasks and analyzing elementary students’ solutions to these tasks	Neither analysis of students’ solutions, nor analysis of open-ended arithmetic tasks
Post-test: diagnostic tasks d_1_ and d_3_ (see Section 2.2; all conditions)			
Experimental phase 2			
	Conditions		
C_1_-task, *n* = 37	C_2_-student, *n* = 40	C_3_-integrated, *n* = 35	
Pre-test: diagnostic tasks d_1_ and d_2_ (all conditions)			
Treatment A Treatment B	Treatment B Treatment A	Treatment AB Treatment AB	
Second post-test: diagnostic tasks d_1_ and d_4_ (all conditions)			

Experimental phase 2 was conducted based on the hypothesis that condition C_3_-integrated, which integrates both treatments A and B in treatment AB, is the optimal condition. Therefore, in condition C3-integrated, we continued with the integrated treatment AB. In condition C_1_-task, prospective teachers were assigned firstly treatment A and, afterwards, treatment B. In condition C_2_-students, prospective teachers were assigned firstly treatment B and, afterwards, treatment A. Thus, in experimental phase 2, we investigated whether the order of the treatment components is significant (RQ2). The design of experimental phase 2 is presented in Table 3. Experimental phase 2 was built upon experimental phase 1, and was based on the same sample apart from the prospective teachers in condition C_0_-control. The whole treatment lasted one semester, i.e., 14 weeks including 90 min of seminars each week (*cf.* Section 2.1).

Both phases of the experiment were carried out in accordance with the University Research Ethics Standards. Participation was voluntary and anonymity was guaranteed.

### 2.3 Measurement

**Figure 4:**
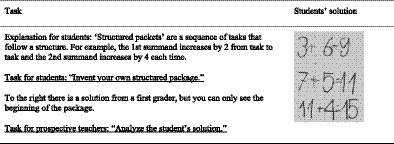
Example of a test-item.

In experimental phase 1, all groups participated in the pre-test at baseline and in the post-test after the 7 weeks of treatment described previously. Referring to experimental phase 2, the three treatment groups additionally participated in a second post-test that took place 1 week after the treatments finished. All three tests contain two written elementary school students’ solutions to open-ended arithmetic tasks. One item remained the same across all tests, but was presented in a slightly different manner (diagnostic item d1). The slight variation is intended to hinder learning effects. Figure 4 shows the item that is included in all three tests (Hirt and Wälti, 2019).

One item changed from test to test (diagnostic items d2, d3 and d4), so a new open-ended arithmetic task with a student’s solution had to be analyzed for each test. These three pre-test tasks and the two post-tests include more extensive students’ solutions to open-ended tasks. These test items are provided in an open repository.^1^

The prospective teachers’ assessments of students’ written solutions were coded through content analyses. The content analysis focused on the epistemic activities and the given codes were categorized by the two facets of the diagnostic thinking process: “perceiving” and “interpreting.” Perceiving addresses the “identification of manifest characteristics,” and “interpreting” comprises the “generation of hypotheses,” and “supported hypotheses” (see Eichler et al., 2022). With this, we counted the number of manifest characteristics, generated hypotheses and supported hypotheses as measurements of prospective teachers’ diagnostic competence in terms of their diagnostic thinking.

In addition, we differentiated perception and interpretation regarding the different content-oriented aspects of a student’s solution: basic arithmetic knowledge, the solution process or the communication of a solution (Rathgeb-Schnierer and Schütte, 2011). Further differentiation of facets of these content-oriented aspects is taken from a previous study (Eichler et al., 2022) and is presented in Table 4.

**Table 4:** Content-oriented areas and facets of prospective teachers’ diagnoses.

Content-oriented area	Basic arithmetic knowledge	Solution process	Communication
Content-oriented facets	Correct calculation Calculation mistakes Place value Transitions Number concept Calculation tricks Number representation	Solution strategies Relations betweentasks Inventing	External shapes Comments Task understanding

Based on the different content-oriented facets, we defined a further measurement: “variety of teachers’ diagnoses.” This variety is provided by the number of different content-oriented facets that were addressed by the prospective teachers referring to the two test items. A previous study revealed that the amount and the variety of epistemic activities are only weakly correlated and thus provide different information about the quality of prospective teachers’ diagnostic thinking (Eichler et al., 2022).

Thus, in the first step of the coding-process, it was ascertained whether a manifest characteristic or a generated hypothesis was present (*cf.* Table 5). A manifest characteristic is identified if the addressed aspect is concretely visible in the student’s solution. A hypothesis is generated if the utterance goes beyond obvious features in the student’s solution and includes interpretation. In the second step, the addressed content-oriented facet was identified. For example, if the statement referred to correct calculation, this code was assigned, while if it referred to the supposed solution strategy, the corresponding code was assigned. In the third and final step, the analysis was examined for supported hypotheses. For this purpose, words were identified that introduce reasoning, such as “because.”

**Table 5:** Exemplary coding of a diagnosis of a prospective teacher.

Example sentence	Associated code
The child calculates all tasks correctly	Manifest characteristic: correct calculation (basic arithmetic knowledge)
The child calculates safely in the number range up to 20	Hypothesis: correct calculation (basic arithmetic knowledge)
The child calculates safely in the number range up to 20 because he/she calculates all tasks correctly	Supported Hypothesis: correct calculation (basic arithmetic knowledge)

The results were evaluated with variance statistics. Mixed ANOVA were calculated, referring to the time (within factor) and the condition (between factor). For both the between and within results, *post hoc* analyses were conducted mainly with t-tests.

## 3 Results

The results section is divided into two parts. In the first part, we present results regarding the first research question (RQ1): How do interventions that address the two phases of diagnostic thinking affect prospective teachers’ epistemic activities? In the second part, we address the second research question (RQ2): Is a specific order addressing the two phases of diagnostic thinking in an intervention more effective in improving prospective teachers’ diagnostic thinking than other orders? In both parts, the number and variety of epistemic activities of prospective teachers are analyzed.

### 3.1 Effects of the interventions on the epistemic activities

**Figure 5:**
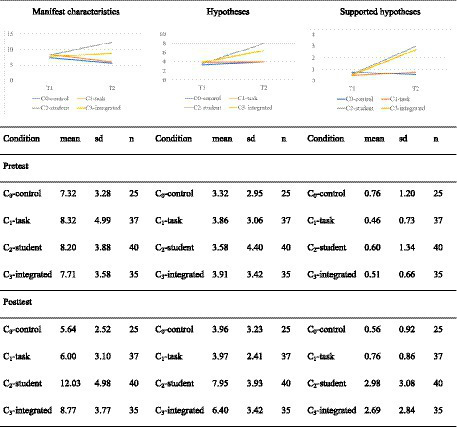
Development of the epistemic activities.

Figure 5 shows the prospective teachers’ development in indicating manifest characteristics, generating hypotheses, and generating supported hypotheses in students’ written solutions from the pre-test to the post-test. For identifying manifest characteristics (left side of Figure 5), a mixed-ANOVA shows a significant interaction between time and condition [*F*(3,133) = 13.145; *p* < 0.01; *η*^2^ = 0.229] with a large effect size. Pairwise t-tests show that the differences between the conditions in the pre-test are not significant (*p* = 1 after Bonferroni correction). This means that prospective teachers in all conditions start at nearly the same level. In the post-test, the pairwise *t*-tests reveal significant differences between condition C_2_-students and all the other conditions (with Bonferroni correction) with mostly large effects (see Table 6 for the test statistics and effect sizes). In addition, significant differences between condition C_3_-integrated and both conditions C_1_-tasks and C_0_-control can be found with large effects, while the conditions C_2_-student and C_3_-integrated do not differ significantly (*p* = 1 after Bonferroni correction).

**Table 6:** Pairwise-*t*-tests at post-test (C_0_-control, C_1_-task, C_2_-student, C_3_-integrated).

	df	*t*	*p*	|*d*|		df	*t*	*p*	|*d*|
Manifest characteristics									
C_2_-C_0_	60.9	6.83	<0.01	1.62	C_3_-C_0_	57.8	3.60	<0.05	1.00
C_2_-C_1_	66.0	−6.42	<0.01	1.45	C_3_-C_1_	65.9	3.39	<0.05	0.81
C_2_-C_3_	71.6	3.21	<0.01	0.74	C_1_-C_0_	–	–	1	–
Generated hypotheses									
C_2_-C_0_	58.2	4.45	<0.01	1.11	C_3_-C_0_	53.6	2.81	<0.05	0.73
C_2_-C_1_	65.4	5.40	<0.01	1.22	C_3_-C_1_	60.7	3.46	<0.01	0.82
C_2_-C_3_	–	–	0.43	–	C_1_-C_0_	–	–	1	–
Supported hypotheses									
C_2_-C_0_	49.3	4.65	<0.01	1.06	C_3_-C_0_	43.4	4.14	<0.01	1.01
C_2_-C_1_	45.6	4.38	<0.01	0.98	C_3_-C_1_	0	3.86	<0.01	0.92
C_2_-C_3_	–	–	1	–	C_1_-C_0_	–	–	1	–
Variety of manifest characteristics									
C_2_-C_0_	61.8	6.16	<0.01	1.51	C_3_-C_0_	54.6	3.90	<0.01	1.01
C_2_-C_1_	70.9	6.16	<0.01	1.40	C_3_-C_1_	68.7	3.75	<0.01	0.88
C_2_-C_3_	2.68	2.68	0.054	0.62	C_1_-C_0_	–	–	1	–
Variety of hypotheses									
C_2_-C_0_	52.1	4.94	<0.01	1.26	C_3_-C_0_	49.8	3.16	<0.01	0.83
C_2_-C_1_	72.2	6.22	<0.01	1.41	C_3_-C_1_	66.0	4.02	<0.01	0.95
C_2_-C_3_	–	–	0.27	–	C_1_-C_0_	–	–	1	–
Variety of supported hypotheses									
C_2_-C_0_	53.5	4.57	<0.01	1.06	C_3_-C_0_	46.4	4.17	<0.01	1.02
C_2_-C_1_	48.8	0.427	<0.01	0.96	C_3_-C_1_	42.1	3.87	<0.01	0.92
C_2_-C_3_	–	–	0.83	–	C_1_-C_0_	–	–	0.4	–

The prospective teachers in condition C_2_-students showed the strongest development. More specifically, the prospective teachers in this condition identified a mean of 8.2 manifest features in the pre-test and 12.0 in the post-test This is a significant difference [*t*(73.6) = −3.83, *p* < 0.01 after Bonferroni correction] with a large effect (Cohen’s |*d*| = 0.86). The amount of identified manifest characteristics of the prospective teachers in condition C_3_-integrated (in which the treatments A and B were mixed) is also increased (not significantly) while for the prospective teachers who only solved open-ended arithmetic tasks (C_1_-task), this amount decreased [*t*(60.2) = 2.41, *p* < 0.05 after Bonferroni correction, Cohen’s |*d*| = 0.56] with a moderate effect. Since the prospective teachers in condition C_0_-control also present a decreasing number of manifest characteristics [*t*(45.0) = 2.03, *p* < 0.05 after Bonferroni correction, Cohen’s |*d*| = 0.58] we assume that the post-test was at least slightly more difficult than the pre-test, due to the exchange of one test item with a new alternative.

For the generation of hypotheses, a mixed-ANOVA shows a significant interaction effect [*F*(3,133) = 9.46, *p* < 0.001] between time and conditions with a large effect size (*η*^2^ = 0.176). Additionally, for the generation of supported hypotheses, a mixed-ANOVA shows a significant interaction effect between condition and time [*F*(3,133) = 13.4, *p* < 0.001] with a large effect size (η^2^ = 0.232). Pairwise t-tests show no significant differences between the four conditions in the number of hypotheses and supported hypotheses generated in the pre-test (*p* = 1 after Bonferroni correction). While the conditions C_2_-student and C_3_-integrated do not differ significantly in the post-test, they show a significant difference from C_1_-tasks and C_0_-control with mostly large effect sizes (see Table 6 for the test statistics and effect sizes).

The prospective teachers in condition C_2_-students showed the strongest development in the generation of hypotheses and supported hypotheses. Prospective teachers in this condition generate a mean of 3.6 hypotheses in the pre-test and a mean of 7.9 hypotheses in the post-test, which is a significant difference [*t*(77.0) = −4.69, *p* < 0.01 after Bonferroni correction] with a large effect (Cohen’s |*d*| = 1.05). Furthermore, they generate a mean of 0.6 supported hypotheses in the pre-test and a mean of 2.98 supported hypotheses in the post test, which is a significant difference [*t*(53.2) = −4.48, *p* < 0.01 after Bonferroni correction] with a large effect (Cohen’s |*d*| = 1). In addition, the development in condition C_3_-integrated is also significant for the generation of hypotheses [*t*(68.0) = −3.04, *p* < 0.01 after Bonferroni correction] with a moderate effect (Cohen’s |*d*| = 0.73) and for the generation of supported hypotheses [*t*(37.7) = −4.41, *p* < 0.01 after Bonferroni correction] with a large effect (Cohen’s |*d*| = 1.05). The developments in conditions C_1_-task and C_0_-control are not significant for the generation of either hypotheses or supported hypotheses.

Regarding all three measured variables, analyzing students’ solutions in isolation (C_2_-student) and in combination with solving open-ended arithmetic tasks (C_3_-integrated) has led to improvements. However, no improvements were achieved by solving open-ended arithmetic tasks in isolation (C_1_-task). For this reason, the order of the effectiveness of the four conditions hypothesized in H1 is only confirmed partly concerning the number of epistemic activities. Actually, condition C_2_-task is at least equally effective as C_3_-integrated. Furthermore, we found no difference between C_1_-task and C_0_-control.

To gain deeper insight into prospective teachers’ diagnostic thinking, we analyzed the number of different competence facets that prospective teachers indicated in students’ written solutions (variety of epistemic activities, see Section 3.3) referring to manifest characteristics, hypotheses and supported hypotheses.

**Figure 6:**
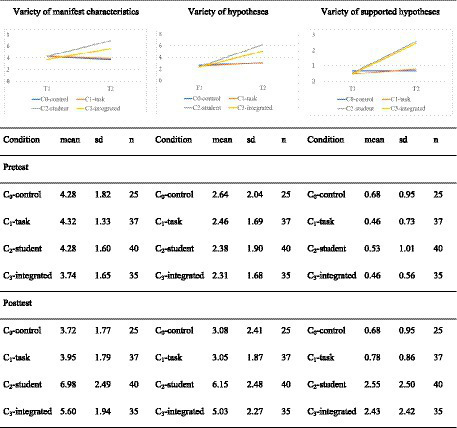
Development of variety of the epistemic activities.

For the variety of manifest characteristics (see Figure 6 on the left side), a mixed-ANOVA shows a significant interaction effect between time and conditions [*F*(3,133) = 18.85, *p* < 0.001] with a large effect size (*η*^2^ = 0.298). Pairwise t-tests show that no significant differences are observable in the pre-test (*p* = 1 after Bonferroni correction). While the results between conditions C_2_-student and C_3_-integrated do not differ significantly in the post-test, they significantly differ from C_1_-tasks and C_0_-control. The difference between C_1_-tasks and C_0_-control is not significant (see Table 6 for the test statistics and effect sizes).

For the development of prospective teachers in the different conditions, it is clear that the variety of manifest characteristics in C_2_-student [*t*(66.6) = −5.78, *p* < 0.01 after Bonferroni correction, Cohen’s |*d*| = 1.29] and in C_3_-integrated [*t*(66.3) = −4.31, *p* < 0.01 after Bonferroni correction, Cohen’s |*d*| = 1.03] increase significantly with large effects but not in C_1_-task and C_0_-control.

For the variety of hypotheses (Figure 6 in the middle) and the variety of supported hypotheses (Figure 6 on the right-hand side) mixed-ANOVAs show significant interaction effects between time and condition [for the variety of hypotheses *F*(3,133) = 17.11, p < 0.001 and for the variety of supported hypotheses *F*(3,133) = 12.87, *p* < 0.001] and large effect sizes (hypotheses: *η*^2^ = 0.279; supported hypotheses: *η*^2^ = 0.225). While the conditions do not differ significantly in the pre-test, in the post-test the conditions again differ significantly, apart from C_2_-student and C_3_-integrated as well as C_1_-task and C_0_-control (see Table 6 for test statistics and effect sizes).

For prospective teachers’ development in the different conditions, we can see that (after Bonferroni correction) the variety of hypotheses and supported hypotheses in C_2_-student [hypotheses: *t*(73.2) = −7.64, p < 0.01, Cohen’s |*d*| = 1.71; supported hypotheses: *t*(51.4) = −4.75, *p* < 0.01, Cohen’s |*d*| = 1.06] and in C_3_-integrated [hypotheses: *t*(62.6) = −5.69, *p* < 0.01, Cohen’s |*d*| = 1.36; supported hypotheses: *t*(37.6) = −4.70, *p* < 0.01, Cohen’s |*d*| = 1.12] increases significantly with large effects while C_1_-task and C_0_-control (once again) do not.

Thus, this additional variety analysis partially confirms hypothesis H1. Again, the effect of order of conditions is not at all as expected, since C_3_-integrated does not outperform C_2_-student and there is no difference observable between C_1_-task and C_0_-control.

### 3.2 Effectiveness of the order of the interventions

**Figure 7:**
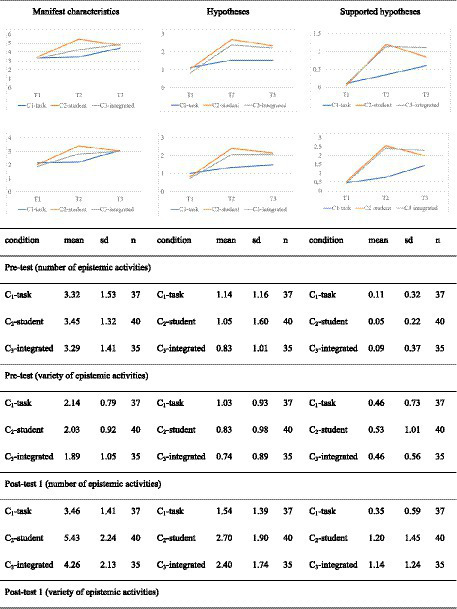

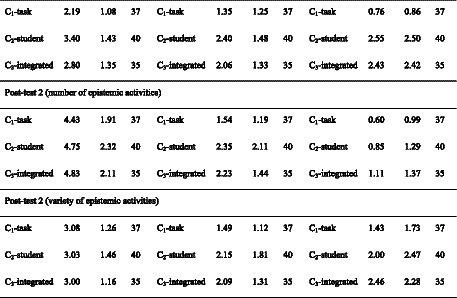
Development of the conditions between all three test dates.

Phase 2 of our experiment addresses the question of whether the order of treatments matters. Here we investigate whether, for example, the prospective teachers who have solved open-ended arithmetic tasks catch up with those who have analyzed solutions and now solve open-ended arithmetic tasks. Since different test items do not seem to present the same level of difficulty (see above), this analysis is restricted to test item d1, which remained the same in every test. Figure 7 shows the development in the groups over the entire semester concerning the three test dates.

Our analyses reveal no significant differences between the conditions in test time T1 and T3 (Table 7).

**Table 7:** Interaction effects of group*time.

Variable	DFn, DFd	*F*	*p*	pes
Number of epistemic activities				
manifest characteristics	2,109	0.54	0.58	0.01
hypotheses	2,109	2.56	0.08	0.05
supported hypotheses	2,109	1.88	0.16	0.03
Variety of epistemic activities				
manifest characteristics	2,109	1.13	0.33	0.02
hypotheses	2,109	2.83	0.06	0.05
supported hypotheses	2,109	1.86	0.16	0.03

We confirmed this by the pairwise t-test with Bonferroni correction. However, the analyses between the test time points for the three conditions indicate significant differences. With two exceptions, the results show significant differences between test times T1 and T3 for the conditions with medium effect sizes [e.g., C_2_-students for the number of manifest characteristics: *t*(61.9) = 3.08; *p* < 0.01; |*d*| = 0.69] to large effect sizes [e.g., C_3_-integrated for the variety of generated hypotheses: *t*(59.6) = 5.01; *p* < 0.01; |*d*| = 1.20]. Both exceptions refer to prospective teachers who started with intervention C_1_-task. For the number of hypotheses generated [*t*(71.9) = 1.48; *p* = 0.14] and for the variety of hypotheses generated [*t*(69.6) = 1.92; *p* = 0.59; |*d*| = 0.44], the differences between pretest and posttest (T3) are not significant. However, the difference for the variety of the generated hypotheses registers as borderline to significant (*p* = 0.059). Regarding the amount and variety of supported hypotheses, there is a tendency for the differences between pretest and posttest for group C_3_-integrated to show larger effect sizes than the differences for group C_2_-students, which in turn show larger effect sizes than the differences for group C_1_-tasks.

Finally, we consider the development in the single conditions from T2 to T3. Thereby, we see that prospective teachers in the C_2_-student group, which received the treatment focusing on student solutions from T1 to T2 and the treatment focusing on tasks from T2 to T3, and the C_3_-integrated prospective teachers, who received the integrated intervention throughout, reveal no significant increase regarding the amount and variety of all epistemic activities. Descriptively, the number and variety of epistemic activities decreases for prospective teachers in condition C_2_-students. However, neither the positive nor the negative changes between test time T2 and test time T3 are significant when subjected to pairwise t-tests. Prospective teachers in C_1_-task, which is the condition that receives the task-focused intervention between T1 and T2 and the student-focused intervention between T2 and T3, show an increase or no change between measurement times T2 and T3. For example, the variety of the identified manifest characteristics at time T2 is on average 2.19, and at time T3 is 3.08, while the number of generated hypotheses at time T2 is 1.54 and in T3 also 1.54. Pairwise t-tests show that the differences between T2 and T3 are partly significant for prospective teachers in condition C_1_-tasks. Significant differences were found for the number of manifest characteristics [t(66.2) = 2.50; *p* < 0.05; |*d*| = 0.58], for the variety of manifest features [t(70.3) = 3.28; *p* < 0.01; |*d*| = 0.76], and for the variety of generated supported hypotheses [*t*(53.0) = 2,13; *p* < 0.05; |*d*| = 0.50). The differences between T2 and T3 for the number of generated hypotheses, the number of supported hypotheses, and the variety of generated hypotheses are not significant, respectively.

When we compare prospective teachers in the three conditions at T3, there are no differences between the conditions in terms of the number and variety of manifest characteristics. Regarding the number and variety of the generated hypotheses, there is no difference between the C_2_-students and C_3_-integrated conditions. However, both groups achieve better results regarding the number and variety of hypotheses than in the C_1_-task. Finally, descriptive results suggest the following hierarchy concerning the number and variety of supported hypotheses: C_3_-integrated > C_2_-students > C_1_-task.

## 4 Discussion

The present study contributes to research on effectively fostering diagnostic thinking as a part of prospective teachers’ diagnostic competence. We investigated diagnostic thinking of prospective teachers in a diagnostic situation involving students’ written solutions to open-ended arithmetic tasks. In this regard, we followed the approach of analyzing diagnostic thinking in authentic tasks for prospective teachers (Südkamp et al., 2012 Enenkiel et al., 2022).

One central idea of this research was focusing on analyzing different conditions for improving prospective teachers’ diagnostic thinking. The crucial idea for the definition of conditions was to differentiate between two phases of diagnostic thinking (Figure 2) according to Nickerson (1999) and, in this regard, to specify the model of diagnostic thinking by Loibl et al. (2020) as proposed by the authors (*cf.* also Ostermann et al., 2018; Philipp, 2018).

Another central idea in modeling and measuring prospective teachers’ diagnostic thinking is to differentiate between epistemic activities regarding perceiving and interpreting. In this respect, we referred to an observation of Stahnke et al. (2016), who revealed a lack of combined analyses of aspects of teachers’ thinking in terms of perceiving and interpreting in their meta-analysis. One approach that combines perceiving and interpreting is used by Enenkiel et al. (2022). In addition to Enenkiel et al. (2022), we differentiated between hypotheses and supported hypotheses, considering the latter as a more elaborated epistemic activity (*cf.* also Fischer et al., 2014; Chernikova et al., 2020). In addition, we distinguished between the number and variety of epistemic activities, assuming that the variety of epistemic activities compared to the number of said activities was a more elaborate measurement of prospective teachers’ diagnostic thinking. The variety of epistemic activities correlated only moderately with the number of epistemic activities in Eichler et al. (2022). However, in this research, the number of epistemic activities and the variety of epistemic activities correlated at least strongly in both post-tests. For this reason, both measurements could potentially be used interchangeably.

In addition, the treatments in all conditions are based on aspects that have previously been proven to promote diagnostic competence (problem-solving and the sub-facets of scaffolding: providing prompts, providing examples, assigning roles, including reflection phases) (Chernikova et al., 2020). We investigated the conditions in 2 experiments, each following a specific research question.

“How do interventions that address the two phases of diagnostic thinking affect prospective teachers’ epistemic activities?” Referring to RQ1, data analyses showed that it is possible to significantly improve prospective teachers’ diagnostic competence with regard to epistemic activities with a treatment that lasts half a semester (7 weeks) and includes students’ solutions.

A sole focus on analyzing students’ solutions in condition C_2_-students was as effective as condition C_3_-integrated, where both phases, i.e., focusing on prospective teachers’ solving of the open-ended tasks and analyzing students’ solutions, were integrated. By contrast, condition C_1_-tasks, including solving the open-ended arithmetic tasks, seems to have no impact on prospective teachers’ diagnostic thinking. The results contribute to existing research about promoting prospective teachers’ diagnostic competence (Klug et al., 2016; Herppich et al., 2018). Regarding the aspect of assigning roles (Chernikova et al., 2020), we can hypothesize that assigning the teacher’s perspective is likely to have a positive effect, whereas solely assigning the role of student in our study seems to have no effect. This result, in particular, was not expected since the open-ended tasks also required some effort from the prospective teachers in order to develop substantial solutions (*cf.* also Brunner et al., 2011). For this reason, we expected that the in-depth analysis of these open-ended tasks would result in heightened awareness by prospective teachers regarding potential solutions and difficulties of these tasks, subsequently leading to enhanced diagnostic thinking. An additional differentiation concerning the quality of epistemic activities, such as the quality of hypotheses about a written student solution, may provide deeper insight into the effect of a prospective teacher’s solution of diagnostic tasks. A further subsequent research question could tackle whether the effect of assigning roles is dependent on the form of tasks, which could be open-ended (*cf.* Besser et al., 2015) or less open-ended (*cf.* Philipp and Gobeli-Egloff, 2022). Finally, a subsequent research question could be whether the effect of assigning roles is dependent on the prospective teachers’ personal characteristics (Loibl et al., 2020), which were found to influence prospective teachers’ diagnostic competence (Chernikova et al., 2020). Because we emphasized epistemic activities such as the identification of manifest characteristics, the generation of hypotheses, the generation of supported hypotheses, and the variety of these epistemic activities, our research contributes to existing research on improving prospective teachers’ diagnostic competence (Chernikova et al., 2020) in terms of diagnostic thinking (Loibl et al., 2020).

Referring to RQ 2, “Is a specific order addressing the two phases of diagnostic thinking in an intervention more effective in improving prospective teachers’ diagnostic thinking than other orders?,” results reveal that a specific order of treatments concerning the two phases of diagnostic thinking adapted from Nickerson (1999) has no effect. In particular, an integrated treatment for both phases did not result in increased diagnostic thinking of prospective teachers. This result in some sense jars with research implying the integration of learning topics is more powerful than a separated and subsequent presentation of different learning topics (Dunlosky et al., 2013; Harr et al., 2015; Ebersbach et al., 2022). Particularly, with respect to the results of RQ 1, it becomes clear that the order of the treatments plays a subordinate role in our research. Thus, the main difference between the conditions seems to be the focus on students’ written solutions, independent of the order of treatments.

Beyond the posed research questions, our results reveal that the first part of our treatment (7 weeks) seems to have generated the main effect. This additional observation is possible when the focus is only on the diagnostic task d1, which remained the same in all three tests. For this task, our results for all experimental conditions (without the control group) reveal significant differences between the pre-test and the post-test with large effects, but a stagnation or even a slight decrease between the initial post-test and second post-test.

Also, beyond our main focus, our results imply a strong influence of the tasks on prospective teachers’ epistemic activities. As expected, the epistemic activities substantially differed between evaluating a student’s solutions concerning the open-ended tasks d2, d3 and d4 on one hand and a student’s solution to task d1 (which was only given partially in the tests) on the other. However, the epistemic activities concerning a student’s solution in the tasks d2, d3, and d4 differed substantially, implying a specific task difficulty for students. For this reason, a closer analysis of prospective teachers’ diagnostic thinking in relation to characteristics of different tasks could be interesting.

### 4.1 Limitations

As outlined previously, the results in this research are related to specific open-ended tasks concerning arithmetic. For this reason, it is an open question of whether and how the results can be replicated with less open-ended tasks, or open-ended tasks in other mathematical subdomains such as calculus. Thus, further research is needed to examine the transferability to other diagnostic situations. Although it has been shown that a treatment which focuses on analyzing student solutions produces good results, its transferability to other situations has not been clarified. Therefore, it needs to be examined whether the influence on the development of diagnostic thinking of self-generating solutions increases for even more demanding tasks.

Another limitation of this study is the quantitative analysis of epistemic activities. Merely counting epistemic activities might obscure the view of possible quality differences, although false and duplicate mentions were excluded.

Finally, person characteristics that we did not regard in this study, such as knowledge or motivational variables, potentially reveal reasons for the effect of the different treatments. In general, the question arises which covariates influence the development of diagnostic thinking. Loibl et al. (2020) include person characteristics in their framework. These include knowledge and motivation but also others, such as stress levels. These are all possible factors that were also addressed by the treatments presented here and thus influence the results. Future research could include these factors and analyze their influence according to the situation.

## 5 Conclusion

The presented study extends previously existing research on the promotion of diagnostic thinking. The design built on previous results and extended those results by explicitly modeling diagnostic thinking with two phases focusing on a diagnostic task and analysis of students’ solutions of the task and, then, specifying the analysis of students’ solutions by epistemic activities. Our results reveal that it is possible to promote prospective teachers’ diagnostic competence particularly if the focus of an intervention is on analyzing student solutions.

## Data availability statement

The raw data are available the conclusions of this article will be made available by the authors, without undue reservation.

## Author contributions

JV: Conceptualization, Investigation, Data curation, Formal analysis, Writing – original draft, Writing - review and editing. AE: Conceptualization, Writing – original draft, Writing - review and editing, Funding aquisition. ER-S: Conceptualization, Writing – original draft, Writing - review and editing, Funding aquisition.

## Footnotes

^1^ https://osf.io/qmfau/

## Appendix 1

Schedule of treatments A, B, and AB.

**Table:**

Week	Treatment A	Treatment B	Treatment AB
Week 1	Introduction to open-ended tasks (prompt)	Introduction to analyzing students’ solutions to open-ended tasks (prompt)	Introduction in open-ended tasks and analyzing students’ solutions to such tasks
Between weeks 1 and 2	Solving task 1	Analyzing a students’ solutions to task 1	Solving task 1
Week 2	Comparing the solutions to task 1 (working in pairs)	Comparing the analyses of students’ solutions to task 1 (working in pairs)	Comparing the solutions to task 1 (working in pairs)
Week 3	Comparing all solutions to task 1 (working as an entire class)	Comparing the analyses of students’ solutions to task 1 (working as an entire class)	Comparing all solutions to task 1 (working as an entire class)
Between weeks 3 and 4	Solving task 2	Analyzing a student’s solutions to task 2	Analyzing two students’ solutions to task 1
Week 4	Comparing the solutions of task 2 (working in pairs)	Comparing the analyses of students’ solutions to task 2 (working in pairs)	Comparing the analyses of students’ solutions to task 1 (working in pairs)
Week 5	Comparing all solutions to task 2 (working as an entire class)	Comparing the analyses of students’ solutions to task 2 (working as an entire class)	Comparing the analyses of students’ solutions to task 1 (working as an entire class)
Between weeks 5 and 6	Solving task 3	Analyzing two students’ solutions to task 3	Solving task 2 and analyzing one student’s solutions to task 2
Week 6	Comparing the solutions to task 3 (working in pairs)	Comparing the analyses of students’ solutions to task 3 (working in pairs)	Comparing own solutions and analyses of a student’s solution to task 2 (working in pairs)
Week 7	Comparing all solutions to task 3 (working as an entire class)	Comparing the analyses of students’ solutions to task 3 (working as an entire class)	Comparing own solutions and analyses of a student’s solutions to task 2 (working as an entire class)
